# Prior treatment with oxaliplatin-containing regimens and higher total bilirubin levels are risk factors for neutropenia and febrile neutropenia in patients with gastric or esophagogastric junction cancer receiving weekly paclitaxel and ramucirumab therapy: a single center retrospective study

**DOI:** 10.1186/s12885-023-11469-y

**Published:** 2023-10-13

**Authors:** Katsuhiko Nara, Takehito Yamamoto, Hiroharu Yamashita, Koichi Yagi, Tappei Takada, Yasuyuki Seto, Hiroshi Suzuki

**Affiliations:** 1 Department of Pharmacy, The University of Tokyo Hospital, 7-3-1 Hongo, Bunkyo-Ku, Tokyo, 113-8655 Japan; 2 The Education Center for Clinical Pharmacy, Graduate School of Pharmaceutical Sciences, The University of Tokyo, 7-3-1 Hongo, Bunkyo-Ku, Tokyo, 113-8655 Japan; 3 Department of Gastrointestinal Surgery, Graduate School of Medicine, The University of Tokyo, 7-3-1 Hongo, Bunkyo-Ku, Tokyo, 113-8655 Japan

**Keywords:** Weekly paclitaxel + ramucirumab, Febrile neutropenia, Oxaliplatin, Total bilirubin, Gastric/Esophagogastric cancer

## Abstract

**Background:**

Weekly paclitaxel + ramucirumab (wPTX + RAM) therapy is recommended as the standard second-line chemotherapy regimen for unresectable advanced/recurrent gastric cancer (GC) or esophagogastric junction cancer. Recent subgroup analysis of the RAINBOW trial revealed a higher frequency of severe neutropenia due to wPTX + RAM in Japanese compared to Western patients. However, no risk factors for severe neutropenia have been identified.

**Methods:**

This retrospective observational study included patients with advanced/unresectable gastric or esophagogastric junction cancer who received wPTX + RAM after failure to respond to platinum and fluoropyrimidine doublet chemotherapy between June 2015 and April 2020. We conducted multivariable logistic regression analyses to identify the risk factors associated with grade 4 neutropenia and febrile neutropenia (FN). In addition, we investigated the relationship between the number of risk factors and overall survival (OS) and progression-free survival (PFS).

**Results:**

Among 66 patients who met the inclusion criteria, grade 4 neutropenia and FN occurred in 21 (31.8%) and 12 (18.2%) patients, respectively. Prior treatment with oxaliplatin-containing regimens was identified as an independent risk factor for developing grade 4 neutropenia (odds ratio (OR) 20.034, 95% confidence interval (95% CI) 3.216–124.807, *P* = 0.001). Total bilirubin of > 1.5 mg/dL (OR 31.316, 95% CI 2.052–477.843, *P* = 0.013) and prior treatment with oxaliplatin-containing regimen (OR 12.502, 95% CI 1.141–137.022, *P* = 0.039) were identified as independent risk factors for developing FN. Next, we classified patients with 0, 1, 2 risk factor(s) as RF-0, RF-1, and RF-2 subgroups, respectively, and compared the PFS and OS among the three subgroups. PFS was not significantly different among the three subgroups, whereas OS was significantly shorter in the RF-2 subgroup (median 1.4 month, 95% CI 0.0–5.3 month) than in the RF-0 subgroup (median 10.2 month, 95% CI 6.8–13.5 month, *P* < 0.01 vs RF-2) and RF-1 subgroup (median 13.3 month, 95% CI 10.9–15.7 month, *P* < 0.01 vs RF-2).

**Conclusions:**

Careful monitoring for grade 4 neutropenia and FN is needed for patients receiving wPTX + RAM therapy who have a history of treatment with oxaliplatin-containing regimens and higher total bilirubin levels.

**Supplementary Information:**

The online version contains supplementary material available at 10.1186/s12885-023-11469-y.

## Background

Gastric cancer (GC) is one of the most common cancers with the fifth highest incidence and fourth highest mortality worldwide [1]. In Japan, it accounts for the second highest morbidity and third highest mortality [2]. Taxanes (paclitaxel (PTX) and docetaxel) and irinotecan (CPT-11) had been used as the standard second-line chemotherapy for unresectable advanced/recurrent GC until 2013 [3]. In 2014, the RAINBOW study showed a significantly improved prognosis for unresectable advanced/recurrent GC in patients who received weekly combination therapy with PTX and ramucirumab (RAM), a monoclonal antibody against vascular endothelial growth factor receptor 2, compared with patients who received PTX monotherapy [4]. Following the RAINBOW trial, weekly PTX + RAM (wPTX + RAM) therapy has been recommended as the standard second-line chemotherapy regimen in the latest GC treatment guidelines [5, 6].

Although it has a favorable efficacy against GC, wPTX + RAM therapy is associated with a high risk of hematologic toxicities, such as leukopenia, neutropenia, and febrile neutropenia (FN) [4]. In addition, a sub-analysis of the RAINBOW study reported by Shitara et al*.* [7] revealed that the risk of hematologic toxicity associated with wPTX + RAM therapy was higher in Japanese patients than in Western patients. Although the underlying mechanisms for this difference have not been examined, it appears to be partially attributable to the higher age of patients in the Japanese subgroup compared to that of the Western subgroup. However, considering that the reported cut-off value of age as a risk factor for FN (≥ 65 years) [8, 9] is higher than the median age of the Japanese subgroup in the RAINBOW study (64.0 years) [7], risk factors other than age may also be involved in the increased risk of FN. Recent studies have revealed that the proportion of older GC patients is increasing in Japan [10, 11]. In addition, in terms of safety, the oxaliplatin (L-OHP)-containing regimen was reported to be superior to the cisplatin-containing regimen for the treatment of unresectable advanced/recurrent GC [12, 13]. However, the cisplatin-containing regimen had been used as a first-line regimen at the time the RAINBOW trial was conducted [14]. In recent clinical practice, an L-OHP-containing regimen has been used as the first-line regimen for unresectable advanced/recurrent GC. Considering these circumstances, patient characteristics and first-line chemotherapy may differ from those in the RAINBOW trial, and it is clinically important to identify the risk factors for FN in the current real-world clinical practice in Japan.

In this study, we aimed to identify the risk factors for developing neutropenia and FN in patients who received wPTX + RAM after failed response to platinum and fluoropyrimidine doublet chemotherapy for unresectable advanced/recurrent GC. We also investigated the relationship between the number of FN risk factors and clinical outcomes (overall survival [OS] and progression-free survival [PFS]).

## Methods

### Patients and treatment

This study included patients with unresectable advanced/recurrent GC or esophagogastric junction cancer who were treated with wPTX + RAM therapy after failed response to platinum and fluoropyrimidine doublet chemotherapy at The University of Tokyo Hospital between June 2015 and April 2020. Patients who received CPT-11 before wPTX + RAM therapy and those lacking clinical data were excluded from the analysis. The wPTX + RAM therapy consisted of a 1-h intravenous infusion of PTX (80 mg/m^2^) on days 1, 8, and 15 and a 1-h intravenous infusion of RAM (8 mg/kg) on days 1 and 15. The cycle length of wPTX + RAM therapy was 28 days, and the therapy was continued until progressive disease (PD) developed or until therapy was ceased due to toxicity or development of adverse effects.

### Data collection and definition

We collected patient characteristics (tumor status, histological types, ECOG performance status (PS), history of surgery, prior platinum and fluoropyrimidine doublet regimens, initial dose, and relative dose intensity (RDI) of wPTX + RAM therapy), laboratory data before wPTX + RAM therapy was started, adverse events, PFS, and OS from electronic medical records. FN was defined as an absolute neutrophil count (ANC) of < 500 cells/µL (or an ANC of < 1,000 cells/µL with an expected decline to < 500 cells/µL in 48 h) and an axillary temperature of ≥ 37.5 °C [15]. Other hematologic and non-hematologic adverse events were evaluated according to the Common Terminology Criteria for Adverse Events version 5.0. The RDI was calculated using the following formula: RDI = Dose intensity/Planned dose intensity × 100 (%), where dose intensity = cumulative dose (mg)/treatment duration (weeks) and planned dose intensity = cumulative planned dose (mg)/planned treatment duration (weeks). PFS was defined as the time from the start of wPTX + RAM therapy to PD (judged by computed tomography, tumor markers, gastrointestinal fiberscopy, and clinical symptoms) or death from any cause. OS was defined as the time from the start of wPTX + RAM therapy to death from any cause. Patients were followed until January 2021.

### Statistical analysis

Continuous and categorical data were expressed as median (range) and percentages, respectively. The Mann–Whitney *U* test was used for continuous data, whereas Fisher’s exact test was used for categorical data. The Kaplan–Meier method was applied to analyze PFS and OS, and a log-rank test was used to analyze the differences in PFS and OS. In addition, we conducted a Cox proportional hazards model analysis to calculate hazard ratios (HRs) of each factor. Bonferroni correction was applied for multiple comparisons. Univariable and multivariable logistic regression analyses were performed to identify risk factors for developing grade 4 neutropenia and FN. In this study, multivariable analysis was performed for factors with *P*-values < 0.10 in the univariable analysis. All tests were two-tailed, and *P*-values < 0.05 were considered statistically significant. All statistical analyses were performed using SPSS Statistics for Windows version 24 (IBM Corp., Armonk, NY, USA).

## Results

### Patient characteristics

A total of 66 patients were included in this study (Table 1). The median age of the study population was 69.1 years old, which was higher than that of the Japanese population in the RAINBOW trial [4]. Prior platinum and fluoropyrimidine doublet regimens included cisplatin (CDDP)-containing regimens (28 patients, 42.4%), and oxaliplatin (L-OHP)-containing regimens (38 patients, 57.6%). RAM was administered at full dose to all patients, whereas the initial dose of PTX was reduced in two patients who received an L-OHP-containing regimen before wPTX + RAM therapy. No patients received prophylactic granulocyte-colony stimulating factor (G-CSF) administration during wPTX + RAM therapy.

**Table 1:** Patient characteristics and laboratory data

Characteristics/laboratory data	*n* = 66
Age [years], median (range)	69.1 (26.5–86.5)
Age ≥ 65 years, n (%)	44 (66.7)
Gender, male (%)	46 (69.7)
ECOG performance status, n (%)	
0	20 (30.3)
1	46 (69.7)
BMI [kg/m^2^], median (range)	19.8 (14.3–25.9)
Tumor status, recurrent (%)	37 (56.1)
History of surgery for primary lesion, n (%)	34 (51.5)
Pathological types, n (%)	
Intestinal type	27 (40.9)
Diffuse type	28 (42.4)
Mix type	9 (13.6)
No data	2 (3.0)
Number of metastatic sites ≥ 2, n (%)	28 (42.4)
Liver metastasis, n (%)	16 (24.2)
Peritoneal metastasis, n (%)	30 (45.5)
Prior fluoropyrimidine-based regimens, n (%)	
CDDP containing regimens	28 (42.4)
L-OHP containing regimens	38 (57.6)
Prior trastuzumab therapy, n (%)	13 (19.7)
PFS of prior fluoropyrimidine-based regimens [months], median (range)	5.6 (1.1–20.8)
Initial dose of wPTX + RAM therapy	
PTX, full dose (%)	64 (97.0)
RAM, full dose (%)	66 (100.0)
Relative dose intensity	
PTX [%], median (range)	72.9 (10.8–100.0)
RAM [%], median (range)	97.6 (39.0–100.0)
Chemotherapy after wPTX + RAM therapy, n (%)	36 (54.5)
Nivolumab, n (%)	25 (37.9)
Irinotecan, n (%)	19 (28.8)
Irinotecan + ramucirumab, n (%)	4 (6.1)
Trifluridine/tipiracil, n (%)	4 (6.1)
Others, n (%)	5 (7.6)
Laboratory data, median (range)	
Alb [g/dL]	3.4 (2.4–4.7)
AST [U/L]	28 (13–105)
ALT [U/L]	15 (5–101)
T-Bil [mg/dL]	0.6 (0.2–2.2)
Cre [mg/dL]	0.70 (0.30–1.41)
Ccr [mL/min]	73.8 (30.9–160.2)
CRP [mg/dL]	0.51 (0.02–14.75)
WBC [count/μL]	5400 (2900–12600)
ANCs [count/μL]	3400 (1000–9300)
Plt [10^4^ count/μL]	17.9 (6.9–44.8)
Hb [g/dL]	11.2 (8.0–15.3)
ALCs [cells/μL]	1200 (400–3300)

*BMI* Body mass index, *CDDP* Cisplatin, *L-OHP* Oxaliplatin, *PFS* Progression-free survival, *PTX* Paclitaxel, *RAM* Ramucirumab, *Alb* serum albumin, *AST* Aspartate transaminase, *ALT* Alanine transaminase, *T-Bil* Total bilirubin, *Cre* Serum creatinine, *Ccr* Creatinine clearance, *CRP* C-reactive protein, *WBC* white blood cell count, *ANCs* Absolute neutrophil counts, *Plt* Platelet count, *Hb* Hemoglobin, *ALCs* Absolute lymphocyte counts

### Adverse events, RDI and risk factors for grade 4 neutropenia/FN

The incidence of adverse events (neutropenia or FN) is summarized in Table 2. Grade 4 neutropenia and FN occurred in 21 (31.8%) and 12 (18.2%) patients, respectively. The incidence of FN in this study was higher than that in the Japanese population enrolled in the RAINBOW trial [4]. In this study population, G-CSF was administrated to approximately 70% (14/21) of patients who developed Grade 4 neutropenia. In addition, 11 out of 12 patients who developed FN received G-CSF administration. Patients who developed grade 4 neutropenia or FN had significantly lower RDI than those without (Table 3).

**Table 2:** Adverse events associated with wPTX + RAM therapy

Adverse events	*n* = 66
Neutropenia ≥ grade 3	36 (54.5)
Neutropenia grade 4	21 (31.8)
Febrile neutropenia ≥ grade 3	12 (18.2)
Thrombocytopenia ≥ grade 3	3 (4.5)
Anemia ≥ grade 3	11 (16.7)
AST increased ≥ grade 3	3 (4.5)
ALT increased ≥ grade 3	1 (1.5)
Nausea ≥ grade 3	0 (0.0)
Vomiting ≥ grade 3	0 (0.0)
Anorexia ≥ grade 3	1 (1.5)
Neuropathy ≥ grade 3	2 (3.0)
Mucositis ≥ grade 3	2 (3.0)
Constipation ≥ grade 3	0 (0.0)
Diarrhea ≥ grade 3	0 (0.0)
Hypertension ≥ grade 3	2 (3.0)
Proteinuria ≥ grade 3	2 (3.0)

Data are shown as n (%)

*AST* Aspartate transaminase, *ALT* Alanine transaminase

**Table 3:** Relative dose intensity in patients with or without grade 4 neutropenia/FN

RDI (%)	With grade 4 neutropenia/FN	Without grade 4 neutropenia/FN	*P-values*
PTX, median (range)	52.7 (10.8–81.3)	83.3 (31.6–100.0)	< *0.001*
RAM, median (range)	100.0 (61.5–100.0)	96.6 (39.0–100.0)	*0.203*

*RDI* Relative dose intensity, *FN* febrile neutropenia

As shown in Table 4, prior treatment with L-OHP-containing regimens (odds ratio (OR) 20.034, 95% confidence interval (95% CI) 3.216–124.807, *P* = *0.001*) was identified as an independent risk factor for developing grade 4 neutropenia after administration of wPTX + RAM therapy. On the other hand, total bilirubin (T-Bil) of > 1.5 mg/dL (OR 31.316, 95% CI 2.052–477.843, *P* = *0.013*) and prior treatment with L-OHP-containing regimens (OR 12.502, 95% CI 1.141–137.022, *P* = *0.039*) were identified as independent risk factors for developing FN after administration of wPTX + RAM therapy (Table 5).

**Table 4:** Univariable and multivariable analysis (grade 4 neutropenia)

Covariates	Univariable analysis		Multivariable analysis	
	OR (95% CI)	*P-values*	OR (95% CI)	*P-values*
Age (≥ 65 years vs. < 65 years)	1.943 (0.602–6.267)	*0.266*		
Gender (female vs. male)	0.426 (0.122–1.486)	*0.181*		
BMI (< 20.0 kg/m^2^ vs. ≥ 20.0 kg/m^2^)	0.333 (0.113–0.987)	*0.047*	0.460 (0.114–1.860)	0.276
Alb (< 3.5 g/dL vs. ≥ 3.5 g/dL)	1.827 (0.598–5.579)	*0.290*		
T-Bil (> 1.5 mg/dL vs. ≤ 1.5 mg/dL)	10.353 (1.079–99.378)	*0.043*	5.668 (0.275–116.974)	*0.261*
Ccr (< 50 mL/min vs. ≥ 50 mL/min)	0.684 (0.126–3.714)	*0.660*		
Prior platinum-based doublet regimens (L-OHP containing vs. CDDP containing)	13.000 (2.698–62.645)	*0.001*	20.034 (3.216–124.807)	*0.001*
No. prior chemotherapy cycles (≥ median vs. < median)	3.400 (1.139–10.147)	*0.028*	3.880 (0.948–15.890)	*0.059*
History of surgery for primary lesion (No vs. Yes)	0.292 (0.096–0.893)	*0.031*	0.267 (0.062–1.143)	*0.075*
Liver metastasis (Yes vs. No)	2.462 (0.784–7.729)	*0.123*		
ANC before wPTX + RAM (< median vs. ≥ median)	0.792 (0.308–2.452)	*0.792*		

*OR* Odds ratio, *CI* confidence interval, *BMI* body mass index, *Alb* Serum albumin, *T-Bil* Total bilirubin, *Ccr* Creatinine clearance, *L-OHP* Oxaliplatin, *ANCs* Absolute neutrophil counts

**Table 5:** Univariable and multivariable analysis (Febrile neutropenia)

Covariates	Univariable analysis		Multivariable analysis	
	OR (95% CI)	*P-values*	OR (95% CI)	*P-values*
Age (≥ 65 years vs. < 65 years)	2.941 (0.585–14.796)	*0.191*		
Gender (female vs. male)	0.400 (0.079–2.022)	*0.268*		
BMI (< 20.0 kg/m^2^ vs. ≥ 20.0 kg/m^2^)	0.400 (0.107–1.490)	*0.172*		
Alb (< 3.5 g/dL vs. ≥ 3.5 g/dL)	3.710 (0.741–18.580)	*0.111*		
T-Bil (> 1.5 mg/dL vs. ≤ 1.5 mg/dL)	26.500 (2.620–268.036)	*0.006*	31.316 (2.052–477.843)	*0.013*
Ccr (< 50 mL/min vs. ≥ 50 mL/min)	1.600 (0.281–9.109)	*0.596*		
Prior platinum-based doublet regimens (L-OHP containing vs. CDDP containing)	11.000 (1.326–91.229)	*0.026*	12.502 (1.141–137.022)	*0.039*
No. of prior chemotherapy cycles (≥ median vs. < median)	2.375 (0.665–8.486)	*0.183*		
History of surgery for primary lesion (No vs. Yes)	0.464 (0.125–1.727)	*0.252*		
Liver metastasis (Yes vs. No)	1.577 (0.408–6.099)	*0.509*		
ANC before wPTX + RAM (< median vs. ≥ median)	0.663 (0.187–2.352)	*0.525*		

*OR* Odds ratio, *CI* Confidence interval, *BMI* Body mass index, *Alb* Serum albumin, *T-Bil* Total bilirubin, *Ccr* Creatinine clearance, *L-OHP* Oxaliplatin, *ANCs* Absolute neutrophil counts

Two risk factors (*i.e.*, T-Bil > 1.5 mg/dL and prior treatment with L-OHP-containing regimens) were identified as independent factors for developing FN. Patients were then classified into three subgroups according to the number of risk factors (*e.g.*, patients with 0, 1, and 2 risk factor(s) were classified into the RF-0, RF-1, and RF-2 subgroups, respectively), and the incidence of FN was compared among the three subgroups. As a result, 27, 35, and 4 patients were classified into the RF-0, RF-1, and RF-2 subgroups, respectively. Among the 35 patients classified into the RF-1 subgroup, 34 patients had a history of prior treatment with L-OHP-containing regimens and 1 patient had T-Bil > 1.5 mg/dL. The incidence of FN increased depending on the number of risk factors (3.7%, 20.0%, and 100% for RF-0, RF-1, and RF-2 subgroups, respectively) (Table 6).

**Table 6:** Incidence of grade 4 neutropenia and FN during wPTX + RAM therapy stratified by the number of risk factors

	RF-0 (*n* = 27)	RF-1 (*n* = 35)	RF-2 (*n* = 4)
Incidence of FN, n (%)	1 (3.7)	7 (20.0)	4 (100.0) ^##, ††^

*RF-0* RF-1, and RF-2 represent the subgroups of the patients with 0, 1, and 2 risk factor(s) for developing grade 4 neutropenia and FN (*i.e.*, T-Bil > 1.5 mg/dL, and prior treatment with oxaliplatin-containing regimens), respectively. *FN* febrile neutropenia

^##^: *P* < *0.01* (RF-0 vs. RF-2)

^††^: *P* < *0.01* (RF-1 vs. RF-2)

### The association of the number of risk factors for FN and PFS/OS

**Fig. 1:**
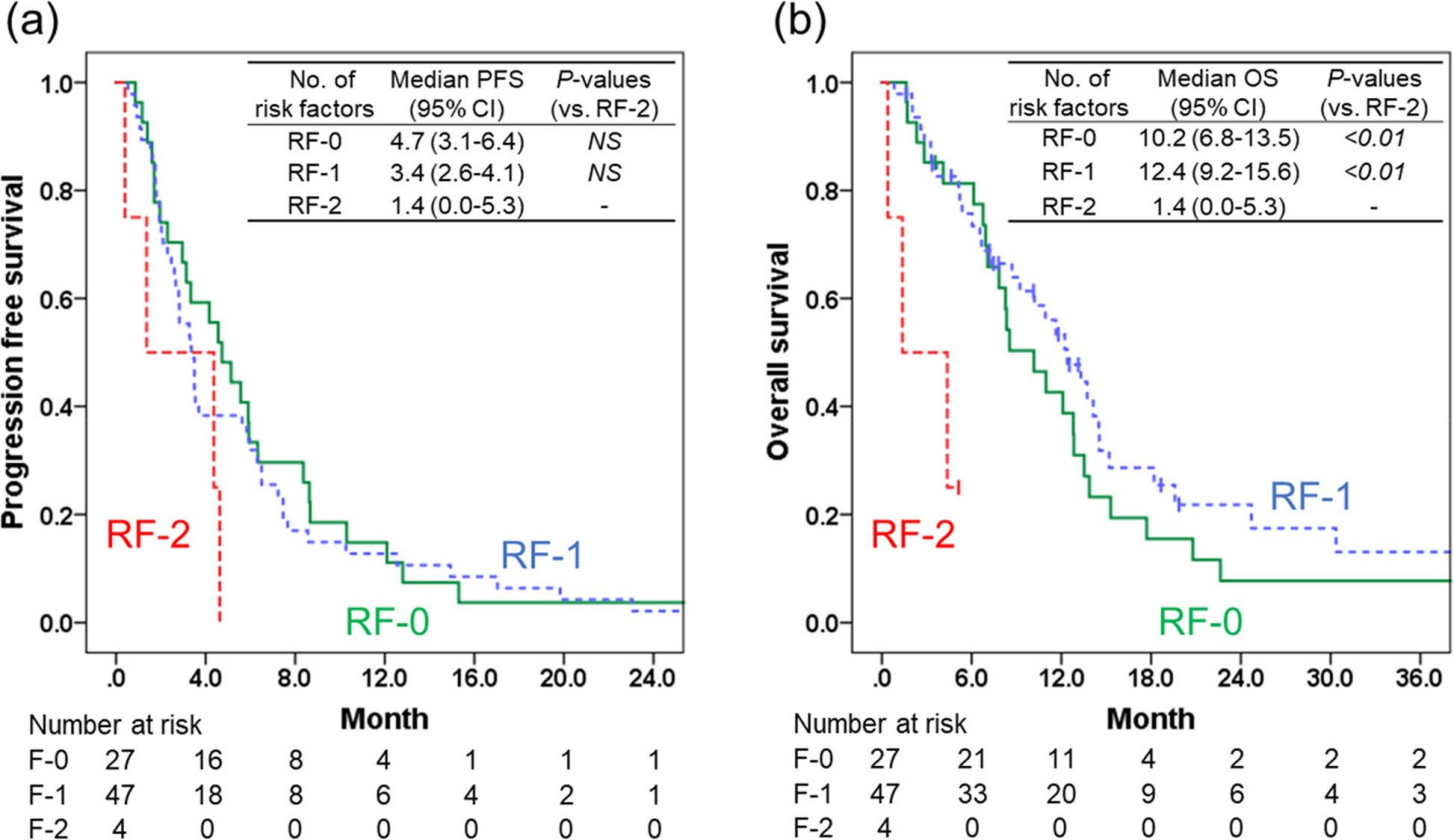
Kaplan–Meier estimates of the overall and progression-free survival after weekly paclitaxel + ramucirumab therapy. Progression-free survival (PFS, panel **a**) and overall survival (OS, panel **b**) after weekly paclitaxel + ramucirumab therapy are shown. RF-0, RF-1, and RF-2 indicate the subgroups of patients with 0, 1, and 2 risk factor(s) for developing FN (total bilirubin > 1.5 mg/dL and prior treatment with oxaliplatin-containing regimens), respectively. The green solid, blue dotted, and red dashed lines represent the RF-0, RF-1, and RF-2 subgroups, respectively. The solid vertical lines represent censored data. A log-rank test with Bonferroni correction was used to analyze differences in PFS and OS

PFS and OS after wPTX + RAM therapy were compared among the three subgroups with different numbers of risk factors (*i.e.*, RF-0, RF-1, and RF-2). As shown in Fig. 1, although no significant differences were observed in PFS among the three subgroups (Fig. 1a), OS was significantly shorter in the RF-2 subgroup than in the RF-0 and RF-1 subgroup (Fig. 1b). In addition, Cox proportional hazard model analysis revealed that RF-0 and RF-1 have significantly lower HR (0.175 [0.046–0.666] and 0.135 [0.035–0.515] for RF-0 and RF-1, respectively) compared with RF-2, indicating that RF-2 is an independent risk factor for OS (Table 7). The other factors included in the Cox proportional hazard model analysis were not significantly associated with OS.

**Table 7:** Cox proportional hazard model analysis for OS

Covariates	Univariable analysis		Multivariable analysis	
	HR (95% CI)	*P-values*	HR (95% CI)	*P-values*
Age (≥ 65 years vs. < 65 years)	0.838 (0.479–1.468)	*0.538*		
Gender (female vs. male)	1.096 (0.823–1.459)	*0.532*		
BMI (< 20.0 kg/m^2^ vs. ≥ 20.0 kg/m^2^)	0.901 (0.523–1.550)	*0.706*		
Alb (< 3.5 g/dL vs. ≥ 3.5 g/dL)	1.529 (0.870–2.689)	*0.140*		
Performance status (1 vs. 0)	1.519 (0.848–2.722)	*0.160*		
History of surgery for primary lesion (No vs. Yes)	0.909 (0.526–1.569)	*0.731*		
Liver metastasis (Yes vs. No)	1.910 (1.024–3.563)	*0.042*	1.755 (0.931–3.310)	*0.082*
The number of risk factors (RF)				
RF = 2	reference	*-*	reference	*-*
RF = 0	0.148 (0.039–0.557)	*0.005*	0.175 (0.046–0.666)	*0.011*
RF = 1	0.113 (0.030–0.426)	*0.001*	0.135 (0.035–0.515)	*0.003*

*HR* Hazard ratio, *CI* Confidence interval, *BMI* Body mass index, *Alb* Serum albumin, *RF* Risk factor

## Discussion

This study revealed that prior treatment with L-OHP-containing regimens and higher T-Bil (> 1.5 mg/dL) are risk factors for developing FN associated with wPTX + RAM therapy after failure to respond to platinum and fluoropyrimidine doublet chemotherapy. In addition, we found that OS was significantly shorter in patients with both risk factors than in those with no risk factors or one risk factor. To the best of our knowledge, this is the first study to identify prior treatment with L-OHP-containing regimens as an independent risk factor for FN development associated with wPTX + RAM therapy.

The incidence of grade ≥ 3 neutropenia in this study was similar to that in the Japanese population in the RAINBOW trial (54.5% vs. 66.2%, respectively) [4]. In contrast, the incidence of FN in this study was higher than that in the Japanese population recruited in the RAINBOW trial (18.2% vs. 4.4%, respectively) [4]. These observations can be attributed to the higher age of patients in this study (median age, 69.1 years vs 64.0 years) (Table 2), considering the previous studies have also reported associations between a higher age with an increased risk of FN. Multivariable logistic regression analysis revealed that prior treatment with L-OHP-containing regimens was an independent risk factor for both grade 4 neutropenia (OR 20.034) and FN (OR 12.502) (Tables 3 and 4). Consistently, when the patients were stratified with platinum agents used in prior platinum-based doublet regimens, the incidence of neutropenia and FN was significantly higher in patients who received L-OHP-containing regimens than in those who received CDDP-containing regimens (Supplementary Table 1).

Although the underlying mechanism for the increased risk of grade 4 neutropenia and FN in patients receiving L-OHP-containing regimens is unclear, it is conceivable that L-OHP-induced sinusoidal obstruction syndrome (SOS) is involved in the increased risk of grade 4 neutropenia and FN. SOS, characterized by the occlusion of centrilobular sinusoidal endothelial cells, is known to lead to liver injury, portal hypertension, splenomegaly, and thrombocytopenia [16–18] and several previous studies have reported that L-OHP is associated with a higher incidence of SOS (51–79%) [19–21]. In contrast, only one case report is available describing SOS associated with CDDP-containing chemotherapy [22], implying that CDDP is associated with a lower incidence of SOS than L-OHP. In addition, an immunohistochemical study by Yoneda et al*.* [18] indicated that the expression level of organic anion transporting polypeptide (OATP) 1B3 in the liver decreases in patients with SOS. As OATP1B3 is known to be a high-affinity hepatocellular transporter of PTX [23] and may affect its pharmacokinetics [23, 24], a decrease in the expression level of OATP1B3 would result in the elevation of serum concentrations of PTX and lead to an increased risk of adverse events. In addition, since SOS is often accompanied by a decrease in platelet counts, we compared the platelet counts and other laboratory data before wPTX + RAM between platinum agents used in prior platinum-based regimens and found that platelet counts were significantly lower in patients who received the L-OHP-containing regimen than in those who received CDDP-containing regimens (Supplementary Table 2), suggesting the occurrence of SOS in patients who received the L-OHP regimen [25, 26].

Higher T-Bil (> 1.5 mg/dL) was also identified as an independent risk factor for the development of FN (OR 22.600) (Table 4). In the RAINBOW trial, a T-Bil of ≤ 1.5 × upper limit of normal (ULN) was set as the administration criterion for the wPTX + RAM regimen at standard dose [4]. Accordingly, because the ULN of T-Bil is set as 1.2–1.5 mg/dL in most hospitals, administration of the wPTX + RAM regimen is acceptable at the standard dose to patients with T-Bil of up to 1.8–2.25 mg/dL according to the criteria in the RAINBOW trial. However, the results of the present study indicate that the risk of developing FN increases even in patients with T-Bil level > 1.5 mg/dL, which is lower than the criterion in the RAINBOW trial. Several previous reports had results consistent with those of our study. A phase I trial by Venook et al. indicated that increased concentrations of PTX and incidences of related toxicities were observed in patients with T-Bil of > 1.5 mg/dL [27]. Furthermore, Joerger et al. recommended a dose reduction of PTX for patients with T-Bil > 1.25 × ULN based on a population pharmacokinetic-pharmacodynamic study [28]. Although the standard dose of PTX was administered to patients with T-Bil of ≤ 1.5 × ULN (up to 1.8–2.25 mg/dL) in the RAINBOW trial, taking these previous reports and our results into consideration, dose reductions of PTX may be required in patients with T-Bil of > 1.5 mg/dL to reduce the risk of FN.

The incidence of FN in the RF-0 subgroup (patients with neither of the two risk factors: prior treatment with L-OHP-containing regimens and T-Bil > 1.5 mg/dL) was 3.7% (1/27). This was similar to that seen in the RAINBOW trial (3%) [4], but the incidence tended to increase with the number of risk factors (Table 5). Notably, in RF-2 (patients with both risk factors), all patients (4/4) experienced FN. Considering that 34 out of 35 patients classified as RF-1 (patients with either of the two risk factors) had history of prior L-OHP treatment as the risk factor, it can be suggested that a history of L-OHP treatment increases the risk of FN after wPTX + RAM therapy to approximately 20%, and that the combination of history of prior L-OHP treatment with T-Bil of > 1.5 mg/dL synergistically increases the risk of FN. This observation appears reasonable because liver dysfunction (most notably, elevated bilirubin) is known as an independent risk factor for developing FN irrespective of chemotherapy regimen [9] and high T-Bil levels may increase the risk of FN after wPTX + RAM therapy by other mechanisms not related to PTX exposure.

Consistently, OS after wPTX + RAM therapy was significantly shorter for RF-2 than for RF-0 and RF-1. This result seems reasonable considering that liver injury is reportedly an independent risk factor for mortality in FN [29]. However, there was no statistically significant difference in PFS among the three subgroups, suggesting that the two risk factors found in our study had a limited impact on the anticancer effect of wPTX + RAM. The elevated T-Bil levels in patients classified in RF-2 group may suggest the biliary obstruction due to liver metastases which is associated with poor OS. Therefore, we examined direct bilirubin levels in patients classified in RF-2 group and found that indirect bilirubin levels were predominantly elevated (Supplementary Table 3). Because biliary obstruction is associated with the direct bilirubin predominant elevation, this observation would suggest that patients in RF-2 group are unlikely developing biliary obstruction associated with liver metastasis. The results of the subgroup analyses suggest that the overall risk of FN associated with wPTX + RAM therapy is low [4]. However, in patients with a history of prior L-OHP treatment, wPTX + RAM therapy is a high-risk regimen for FN. Furthermore, in combination with a high T-Bil (> 1.5 mg/dL), a history of prior L-OHP treatment further synergistically increases the risk of FN and results in a poor prognosis. An important clinical implication from these findings is that more appropriate management of FN may be achieved by stratifying patients based on the two risk factors (i.e., prior L-OHP treatment and T-Bil of > 1.5 mg/dL) before administration of wPTX + RAM therapy [29].

This study has several limitations. First, this was a single-center retrospective study, and it is unknown whether our results are applicable to other facilities. Further studies involving other facilities are required to confirm the generalizability of our results. Second, this study included only Japanese patients. Thus, the frequency of adverse effects in Western countries remains unknown. Studies involving patients abroad are needed to clarify the effects of race, prior treatment, and liver dysfunction on adverse effects. Third, we were unable to measure serum concentrations of PTX. Further studies are required to clarify whether L-OHP affects the pharmacokinetics of PTX, by prospectively measuring the serum concentrations of PTX.

## Conclusions

This is the first study to reveal that prior treatment with L-OHP-containing regimens and higher T-Bil (> 1.5 mg/dL) are independent risk factors for grade 4 neutropenia and FN associated with wPTX + RAM therapy. In addition, patients with both risk factors were at an increased risk of FN and had shorter OS compared to patients with no or one of the two risk factors. Patients with a higher T-Bil and a history of treatment with an L-OHP-containing regimen should be closely monitored for grade 4 neutropenia and FN when receiving wPTX + RAM therapy.

### Supplementary Information

**Additional file 1.**

**Additional file 2.**

**Additional file 3.**

## Data Availability

The datasets used and/or analyzed during the current study are available from the corresponding author upon reasonable request.

## Abbreviations

GC: Gastric cancer
PTX: Paclitaxel
CPT-11: Irinotecan
RAM: Ramucirumab
wPTX + RAM: Weekly paclitaxel plus ramucirumab
FN: Febrile neutropenia
L-OHP: Oxaliplatin
OS: Overall survival
PFS: Progression-free survival
PD: Progressive disease
PS: Performance status
RDI: Relative dose intensity
ANC: Absolute neutrophil count
CDDP: Cisplatin
G-CSF: Granulocyte-colony stimulating factor
OR: Odds ratio
CI: Confidence interval
T-Bil: Total bilirubin
SOS: Sinusoidal obstruction syndrome
OATP: Organic anion transporting polypeptide
ULN: Upper limit of normal
