# Supplementary material for: Prior treatment with oxaliplatin-containing regimens and higher total bilirubin levels are risk factors for neutropenia and febrile neutropenia in patients with gastric or esophagogastric junction cancer receiving weekly paclitaxel and ramucirumab therapy: a single center retrospective study
Source: BMC Cancer. 2023 Oct 13;23:979. doi: 10.1186/s12885-023-11469-y (PMC10571405; doi:10.1186/s12885-023-11469-y)
Supplement: Supplementary file 1 — Additional file 1. [file 12885_2023_11469_MOESM1_ESM.docx]

**Supplementary Table 1.** Incidences of adverse events associated with weekly paclitaxel + ramucirumab therapy stratified by prior platinum-based doublet regimens.

| Adverse events | Platinum agents used in prior platinum-based doublet regimens | | *P*-values |
| --- | --- | --- | --- |
|  | CDDP (n=28) | L-OHP (n=38) |  |
| Neutropenia ≥ grade 3 | 7 (25.0) | 29 (76.3) | <0.001 |
| Neutropenia grade 4 | 2 (7.1) | 19 (50.0) | <0.001 |
| Febrile neutropenia ≥ grade 3 | 1 (3.6) | 11 (28.9) | 0.009 |
| Thrombocytopenia ≥ grade 3 | 0 (0.0) | 3 (7.9) | 0.256 |
| Anemia ≥ grade 3 | 5 (17.9) | 6 (15.8) | 1.000 |
| AST increased ≥ grade 3 | 0 (0.0) | 3 (7.9) | 0.256 |
| ALT increased ≥ grade 3 | 0 (0.0) | 1 (2.6) | 1.000 |
| Nausea ≥ grade 3 | 0 (0.0) | 0 (0.0) | - |
| Vomiting ≥ grade 3 | 0 (0.0) | 0 (0.0) | - |
| Anorexia ≥ grade 3 | 0 (0.0) | 1 (2.6) | 1.000 |
| Neuropathy ≥ grade 3 | 0 (0.0) | 2 (5.3) | 0.504 |
| Mucositis ≥ grade 3 | 0 (0.0) | 2 (5.3) | 0.504 |
| Constipation ≥ grade 3 | 0 (0.0) | 0 (0.0) | - |
| Diarrhea ≥ grade 3 | 0 (0.0) | 0 (0.0) | - |
| Hypertension ≥ grade 3 | 2 (7.1) | 0 (0.0) | 0.176 |
| Proteinuria ≥ grade 3 | 2 (7.1) | 0 (0.0) | 0.176 |

Data are shown as n (%).

CDDP, cisplatin; L-OHP, oxaliplatin; AST, aspartate transaminase; ALT, alanine transaminase.
