# Supplementary material for: Prior treatment with oxaliplatin-containing regimens and higher total bilirubin levels are risk factors for neutropenia and febrile neutropenia in patients with gastric or esophagogastric junction cancer receiving weekly paclitaxel and ramucirumab therapy: a single center retrospective study
Source: BMC Cancer. 2023 Oct 13;23:979. doi: 10.1186/s12885-023-11469-y (PMC10571405; doi:10.1186/s12885-023-11469-y)
Supplement: Supplementary file 2 — Additional file 2. [file 12885_2023_11469_MOESM2_ESM.docx]

**Supplementary Table 2.** Patient characteristics and laboratory data before weekly paclitaxel + ramucirumab therapy stratified by prior platinum-based doublet regimens.

| Characteristics/  Laboratory data | Platinum agents used in prior platinum-based  doublet regimens | | *P*-values |
| --- | --- | --- | --- |
|  | CDDP (n=28) | L-OHP (n=38) |  |
| Age [years], median (range) | 69.7 (26.5-81.5) | 68.5 (30.8-86.5) | 0.697 |
| BMI [kg/m^2^], median (range) | 19.4 (14.7-25.9) | 20.5 (14.3-25.9) | 0.805 |
| Gender, male (%) | 16 (57.1) | 30 (78.9) | 0.057 |
| ECOG PS=0, n (%) | 11 (39.3) | 9 (23.7) | 0.173 |
| Pathological types, n (%) |  |  | 0.543 |
| Intestinal type, n (%) | 11 (39.3) | 16 (42.1) |  |
| Diffuse type, n (%) | 12 (42.9) | 16 (42.1) |  |
| Mix type, n (%) | 5 (17.9) | 4 (10.5) |  |
| No data, n (%) | 0 (0.0) | 2 (5.3) |  |
| Number of metastatic sites ≥ 2, n (%) | 12 (42.9) | 16 (42.1) | 0.951 |
| Liver metastasis, n (%) | 7 (25.0) | 9 (23.7) | 0.902 |
| Peritoneal metastasis, n (%) | 13 (46.4) | 17 (44.7) | 0.891 |
| PFS of prior fluoropyrimidine-based regimens [months], median (range) | 6.4 (1.1-20.8) | 5.2 (1.7-17.5) | 0.414 |
| Initial dose of wPTX + RAM therapy |  |  |  |
| PTX, full dose (%) | 28 (100.0) | 36 (94.7) | 0.504 |
| RAM, full dose (%) | 28 (100.0) | 38 (100.0) | - |
| Relative dose intensity of wPTX + RAM therapy |  |  |  |
| PTX [%], median (range) | 92.7 (45.6-100.0) | 62.8 (10.8-100.0) | <0.001 |
| RAM [%], median (range) | 96.1 (66.9-100.0) | 99.0 (39.0-100.0) | 0.805 |
| Chemotherapy after wPTX + RAM therapy, n (%) | 12 (42.9) | 24 (63.2) | 0.102 |
| Nivolumab, n (%) | 6 (21.4) | 19 (50.0) |  |
| Irinotecan, n (%) | 10 (35.7) | 9 (23.7) |  |
| Irinotecan + ramucirumab, n (%) | 1 (3.6) | 3 (7.9) |  |
| Trifluridine/tipiracil, n (%) | 1 (3.6) | 3 (7.9) |  |
| Others, n (%) | 1 (3.6) | 4 (10.5) |  |
| Laboratory data, median (range) |  |  |  |
| Alb [g/dL] | 3.6 (2.4-4.7) | 3.3 (2.4-4.4) | 0.075 |
| AST [U/L] | 23 (13-44) | 32 (15-105) | <0.001 |
| ALT [U/L] | 13 (5-39) | 17 (7-101) | 0.025 |
| T-Bil [mg/dL] | 0.5 (0.2-1.8) | 0.7 (0.3-2.2) | 0.019 |
| Cre [mg/dL] | 0.74 (0.40-1.18) | 0.70 (0.30-1.41) | 0.595 |
| Ccr [mL/min] | 74.6 (30.9-160.2) | 72.7 (35.4-149.5) | 0.697 |
| CRP [mg/dL] | 0.35 (0.02-9.64) | 0.71 (0.02-14.75) | 0.559 |
| WBC [/μL] | 5700 (3200-12600) | 5100 (2900-10500) | 0.112 |
| ANCs [/μL] | 3900 (1600-9300) | 3100 (1000-8500) | 0.082 |
| Plt [10^4^/μL] | 22.9 (6.9-44.8) | 15.2 (9.2-39.1) | 0.002 |
| Hb [g/dL] | 10.4 (8.4-14.7) | 11.5 (8.0-15.3) | 0.391 |
| ALCs [/μL] | 1200 (600-3300) | 1100 (400-2800) | 0.736 |

CDDP, cisplatin; L-OHP, oxaliplatin; BMI, body mass index; PS, performance status; PFS, progression-free survival; PTX, paclitaxel; RAM, ramucirumab; Alb, serum albumin; AST, aspartate transaminase; ALT, alanine transaminase; T-Bil, total bilirubin; Cre, serum creatinine; Ccr, creatinine clearance; CRP, C-reactive protein; WBC, white blood cell count; ANCs, absolute neutrophil counts; Plt, platelet count; Hb, hemoglobin; ALCs, absolute lymphocyte counts.
