# Supplementary material for: Prior treatment with oxaliplatin-containing regimens and higher total bilirubin levels are risk factors for neutropenia and febrile neutropenia in patients with gastric or esophagogastric junction cancer receiving weekly paclitaxel and ramucirumab therapy: a single center retrospective study
Source: BMC Cancer. 2023 Oct 13;23:979. doi: 10.1186/s12885-023-11469-y (PMC10571405; doi:10.1186/s12885-023-11469-y)
Supplement: Supplementary file 3 — Additional file 3. [file 12885_2023_11469_MOESM3_ESM.docx]

**Supplementary Table 3.** Total and direct bilirubin levels of patients in RF-2 group

|  | Total bilirubin [mg/dL] | Direct bilirubin [mg/dL] |
| --- | --- | --- |
| Patient 1 | 1.8 | NA |
| Patient 2 | 2.0 | 0.8 |
| Patient 3 | 2.0 | 0.6 |
| Patient 4 | 2.0 | 0.9 |
| Patient 5 | 2.2 | 0.7 |

NA, not available.
